# An unusual association of three autoimmune disorders: celiac disease, systemic lupus erythematosus and Hashimoto’s thyroiditis

**DOI:** 10.1007/s13317-016-0079-9

**Published:** 2016-07-06

**Authors:** Viera Boccuti, Antonio Perrone, Alessia D’Introno, Anna Campobasso, Moris Sangineto, Carlo Sabbà

**Affiliations:** Department of Interdisciplinary Medicine, University of Bari Aldo Moro, Piazza Giulio Cesare 11, 70124 Bari, Italy

**Keywords:** Multiple autoimmune syndrome, Celiac disease, Systemic lupus erythematosus, Hashimoto’s thyroiditis

## Abstract

Autoimmune disorders are known to be more frequent in women and often associated each others, but it is rare to see multiple autoimmune diseases in a single patient. Recently, the concept of multiple autoimmune syndrome has been introduced to describe patients with at least three autoimmune diseases. We describe a case of a young man with a clinical history of psychiatric symptoms and celiac disease (CD) who was diagnosed to have other two autoimmune disorders: systemic lupus erythematosus (SLE) and Hashimoto’s thyroiditis. This case is unusual upon different patterns: the rare combination of the three autoimmune diseases, their appearance in a man and the atypical onset of the diseases with psychiatric symptoms likely to be related either to CD or to SLE.

## Introduction

Celiac disease (CD) is a genetically linked autoimmune disorder and its prevalence is estimated to be ~1 % in western countries [1]. The association between CD and other autoimmune disorders has been clearly established. In fact, a significantly increased prevalence of other autoimmune diseases has been reported in subjects with CD, with an estimated burden of up to 15 % [2]. However, even for autoimmune endocrine diseases, such as autoimmune thyroid disorders, a coexistence of CD has been widely reported, only some case reports have suggested the association between CD and systemic lupus erythematosus (SLE). SLE is a chronic inflammatory autoimmune disease more prevalent in women that can potentially affect any organ and system and is characterized by a broad range of clinical manifestation. The concept of multiple autoimmune syndrome (MAS) has been introduced to describe patients with at least three autoimmune diseases [3, 4]. MAS can be classified into three groups according to the prevalence of their association with one another: Type 1 comprises myasthenia gravis, thymoma, polymyositis and giant cell myocarditis; Type 2 includes Sjögren’s syndrome, rheumatoid arthritis, primary biliary cirrhosis, scleroderma and autoimmune thyroid disease and Type 3 groups together autoimmune thyroid disease, myasthenia and/or thymoma, Sjögren’s syndrome, pernicious anemia, idiopathic thrombocytopenic purpura, Addison’s disease, insulin-dependent diabetes, vitiligo, autoimmune hemolytic anemia, systemic lupus erythematosus and dermatitis herpetiformis [3]. Herein we describe a case of young man with CD who was diagnosed to be suffering from other two autoimmune conditions: SLE and Hashimoto’s thyroiditis (HT).

## Case report

A 39-year-old man was admitted to this internal medicine unit in February 2015 with a 2-week history of fatigue and fever. The patient reported pain in large joints as associated symptom. He had a clinical history of psychiatric symptoms started when he was 18 and biopsy proven CD diagnosed 6 months before the hospital admission. On general examination he presented malar rash, edema of lower extremities and fever (38 °C). Abdominal, cardiac, pulmonary and neurological examinations were normal. Laboratory data showed hypochromic anemia with hemoglobin value of 9.3 g/dl and positive direct Coombs test, an increased erythrocyte sedimentation rate (ESR) of 120 mm/h and a C reactive protein (CRP) of 53 mg/dl; elevated serum levels of total proteins (9.1 g/dl) with hypergammaglobulinemia (42.4 %), decreased C3 and C4 complement factors concentrations and slightly elevation of AST (90 UI/dl); renal function, electrolytes, glycaemia, blood coagulation tests, tumor markers were within normal levels. The antinuclear antibodies were positive at 1:1280 as well as anti-dsDNA and anti-SSA were positive at >240 and >640, respectively; rheumatoid factor, anti-Sm, anti RNP, and anti-SSB were negative. Thyroid profile showed increased thyroid stimulating hormone (TSH) 7.34 mUI/l (n.v. = 0.3–3.6), low FT4 0.7 ng/dl (*N* = 0.9–1.7) and low FT3 1.8 pg/ml (*N* = 2.2–4.2); both serum thyroglobulin and antiperoxidase antibodies were positive and were >100 and >150, respectively. Ultra sound scan showed non toxic goiter characterized by diffuse enlarged gland and heterogeneous echotexture. Serological testing for syphilis, HIV Type 1 and 2, HSV Type 1 and 2, CMV, EBV, influenza, adenovirus, and mycoplasma were all negative. Blood and urine cultures were also negative. Based on clinical and laboratory testing a diagnosis of HT and SLE was made. Because the patient was already suffering for CD, we made a final diagnosis of MAS. Methylprednisolone was started for SLE and levothyroxine therapy for HT, with good clinical and laboratory initial response. The patient was advised to continue a strictly gluten free diet for CD.

## Discussion

Disorders of autoimmune pathogenesis occur with increased frequency in patients with a history of another autoimmune disease. The causes for the onset and manifestation of associated diseases are various: genetic predisposition, shared pathogenic mechanisms, and others are of unknown nature [5]. A subject with one autoimmune disease has 25 % chance to get another autoimmune disease [6]. In our case the patient suffering from CD was diagnosed to have other two different autoimmune diseases (SLE and HT). Evidences show that in patients with CD occurrence of thyroid dysfunction is up to 10 % of cases, and risk of thyroid disease is threefold higher as compared to controls [7]; on the other hand, the prevalence of CD is higher in patients with autoimmune thyroid disease, with a prevalence ranging from 1.2 to 9.3 % [7–9]. Similarly, thyroid disease appears to be more frequent in SLE patients than in the general population. Studies reported that SLE is associated to hypothyroidism with a range of variability from 4 to 21 % and patients diagnosed for HT are more prone to develop SLE than normal controls [10]. An association between CD and SLE has been recorded in some case reports [11, 12], with a recent study suggesting that individuals with CD are at threefold increased risk of SLE, but absolute risk is low [11]. Furthermore, it has been shown that SLE may develop later in the clinical course of CD even after a small bowel biopsy response to gluten free therapy [13]. However, to the authors’ knowledge, only one study reported a case of MAS in a young girl suffering from CD, HT and SLE [14]. It is interesting that in our case the association among the three autoimmune diseases was observed in a young man, being the autoimmune disorders more prevalent in women (including CD and SLE) [15]. Moreover, the patient had a clinical history of psychiatric symptoms presented as he was 18 years old that could be likely considered as an early manifestation of SLE. It is known that SLE may cause a broad range of neurological and psychiatric symptoms that can precede the onset of lupus or occur at any time during the course of the disease. However, the psychosis is not a common feature in SLE, and the reported prevalence of lupus psychosis varies from 0 to 11 %. Recently, the diagnosis of lupus psychosis at the moment of the onset of the disease has been described in one-third of the cases of a large series of SLE patients, but it has been observed that it usually occurs within the context of florid activity of the disease, mainly associated with cutaneous or haematological manifestations [16]. It has also to be stressed that several psychiatric symptoms and disorders, such as anxiety disorders, depressive and mood disorders, attention deficit hyperactivity disorder (ADHD) and schizophrenia [17] have been associated with CD, even if the research on the relationship of these psychiatric disorders to CD is limited. On this view, the psychosis in our patient could also be an early manifestation of the CD rather than of SLE. In conclusion, our case report is of interest upon different notes. First, the rare association of the three autoimmune disorders described: CD has been reported as part of MAS in association with HT and SLE just once in literature and is not included in the classification of MAS, so that our patient can be classified only partially within MAS Type 3; second, the appearance of these three autoimmune diseases in a man and finally the atypical onset of the diseases with psychiatric symptoms that can be related either to CD or to SLE. Moreover, this case of MAS highlights the importance to make a good clinical surveillance in patients with one autoimmune disorder because they have a higher risk of developing another autoimmune disease even rarely associated with the first one.

## References

[1] Dubé C, Rostom A, Sy R (2005). The prevalence of celiac disease in average-risk and at-risk Western European populations: a systematic review. Gastroenterology.

[2] Neuhausen SL, Steele L, Ryan S (2008). Co-occurrence of celiac disease and other autoimmune diseases in celiacs and their first-degree relatives. J Autoimmun.

[3] Humbert P, Dupond JL (1988). Multiple autoimmune syndromes. Ann Med Interne (Paris).

[4] Anaya JM, Corena R, Castiblanco J (2007). The kaleidoscope of autoimmunity. Multiple autoimmune syndromes and familial autoimmunity. Expert Rev Clin Immunol.

[5] Alarcon-Segovia D (2004). Shared autoimmunity: the time has come. Curr Rheumatol Rep.

[6] Mohan MP, Ramesh TC (2003). Multiple autoimmune syndrome. Indian J Dermatol Venereol Leprol.

[7] Lauret E, Rodrigo L (2013). Celiac disease and autoimmune-associated conditions. Biomed Res Int.

[8] Teixeira LM, Nisihara R, Utiyama SR (2014). Screening of celiac disease in patients with autoimmune thyroid disease from Southern Brazil. Arq Bras Endocrinol Metabol.

[9] Ventura A, Ronsoni MF, Shiozawa MB (2014). Prevalence and clinical features of celiac disease in patients with autoimmune thyroiditis: cross-sectional study. Sao Paulo Med J.

[10] Lin Wen-Ya, Chang Chia-Li, Lin-Shien Fu (2015). Systemic lupus erythematosus and thyroid disease: a 10-year study. J Microbiol Immunol Infect.

[11] Ludvigsson JF, Rubio-Tapia A, Chowdhary V (2012). Increased risk of systemic lupus erythematosus in 29,000 patients with biopsy-verified celiac disease. J Rheumatol.

[12] Crişcov GI, Stana AB, Ioniue IK (2015). Coexistence of celiac disease and systemic lupus erythematosus in a 6-year-old girl-case report. Rev Med Chir Soc Med Nat Iasi.

[13] Freeman HJ (2008). Adult celiac disease followed by onset of systemic lupus erythematosus. J Clin Gastroenterol.

[14] Latif S, Jamal A, Memon I (2010). Multiple autoimmune syndrome: Hashimoto’s thyroiditis, coeliac disease and systemic lupus erythematosus (SLE). J Pak Med Assoc.

[15] Pennell LM, Galligan CL, Fish EN (2012). Sex affects immunity. J Autoimmun.

[16] Pego-Reigosa JM, Isenberg DA (2008). Psychosis due to systemic lupus erythematosus: characteristics and long-term outcome of this rare manifestation of the disease. Rheumatology.

[17] Jackson JR, Eaton WW, Cascella NG (2012). Neurologic and psychiatric manifestations of celiac disease and gluten sensitivity. Psychiatr Q.

